# Nuclear Receptors and Stress Response Pathways Associated with the Development of Oral Mucositis Induced by Antineoplastic Agents

**DOI:** 10.3390/ph17081086

**Published:** 2024-08-20

**Authors:** Moena Kagaya, Yoshihiro Uesawa

**Affiliations:** Department of Medical Molecular Informatics, Meiji Pharmaceutical University, Tokyo 204-8588, Japan

**Keywords:** oral mucositis, antineoplastic agent, molecular initiating event

## Abstract

Oral mucositis (OM) is one of the common adverse events associated with cancer treatment that decreases the quality of life and affects treatment outcomes. However, the medications used to manage OM are generally only palliative, and our knowledge of the syndrome is limited. The etiology of the syndrome is thought to be complex and multifactorial. We investigated the trends and characteristics of OM and estimated molecular initiating events (MIEs) associated with the development of the syndrome using the FDA Adverse Event Reporting System. The study of trends and characteristics suggested that OM is significantly more likely to occur in females and nonelderly patients and is likely to be induced by protein kinase inhibitors such as afatinib and everolimus. Next, we used Toxicity Predictor, an in-house quantitative structure–activity relationship system, to estimate OM-associated MIEs. The results revealed that the agonist activity of the human pregnane X receptor, thyroid-stimulating hormone-releasing hormone receptor, and androgen receptor may be associated with OM development. Our study findings are expected to help avoid the risk of OM induction during the drug discovery process and clinical use of antineoplastic agents.

## 1. Introduction

In stomatitis, oral mucositis (OM) refers to redness, erosions, and ulcerative lesions in the oral mucosa due to chemotherapy and radiation therapy in cancer treatment [1,2]. The oral mucosa is sensitive to chemotherapy and radiation therapy because oral mucosa cells regenerate quickly. OM is one of the most commonly observed side effects of cancer treatment, estimated to occur in 30–40% of the patients with cancer treated with chemotherapy [3,4,5]. OM is often accompanied by pain and causes a decline in important functions such as speaking and swallowing, leading to poor nutrition, reduced quality of life, and increased risk of infection [6,7]. Decreased treatment outcomes and increased financial burden are consequences of OM [6,8]. Although OM can be a clinically significant adverse event, there is no established cure, and drugs used for management are typically only palliative [9].

Regardless of treatment modalities, such as chemotherapy or radiation therapy, the pathways leading to OM are mostly the same [5], and the etiology of OM is complex and multifactorial [10]. The etiology of OM is based on the five-step theory proposed by Sonis et al., believed to occur in the following order: initiation, damage response, signal transduction and amplification, ulcer formation, and healing [4,10,11,12]. During the initiation phase, cells and DNA are damaged by chemotherapy and radiation therapy, producing a large number of reactive oxygen species in the cytoplasm [10,13]. During the damage response phase, a series of biological events activate several signaling pathways [5]. Nuclear factor-κB (NF-κB) is one of the most prominently expressed pathways activated [14]. In the signal transduction amplification phase, inflammation-inducing cytokines are overproduced to amplify damage [14]. Once a certain level of damage is reached, ulcers with inflammation appear and the epithelial barrier is broken down [4,10]. Ulcers develop in response to tissue damage and secondary infection, along with leukocyte infiltration [14]. Finally, basal epithelial cells move and multiply, healing the ulcer [10].

Although many factors contribute to adverse events, from a toxicological perspective, molecular initiating events (MIEs) in adverse outcome pathways are a key concept [15]. Adverse outcome pathways are defined as a series of events beginning with MIEs [16]. It is a conceptual framework that systematically organizes biologically reliable and empirically supported cause-and-effect correlations from the initial molecular-level changes that a chemical causes in a biological system to adverse outcomes [17]. MIEs are the first step in adverse outcome pathways and are defined as the first interaction between a molecule and a biomolecule or biological system [18]. Its targets include the nuclear receptor (NR) and stress response (SR) pathways. Understanding this process is important in toxicity assessment and risk evaluation [18]. In this study, we investigated the NR and SR pathways targeted by the Tox21 program to understand the MIEs associated with OM development. The Tox21 program utilizes in vitro high-throughput screening technology to evaluate chemical effects on specific biological pathways and includes approximately 10,000 compounds [19].

Although OM is a clinically significant adverse event, there are no established treatments, and our knowledge of OM is limited. MIEs associated with OM development can become new targets for the prediction and management of OM, and the estimation of MIEs may lead to OM risk avoidance in the drug discovery process and clinical use of antineoplastic agents. This study aimed to evaluate OM-associated NR and SR pathways using Toxicity Predictor [20], a quantitative structure–activity relationship (QSAR) system developed in our laboratory based on the measured agonist and antagonist activities of compounds against NR and SR published in the Tox21 program. In addition, this study aimed to identify factors that contribute to OM development.

## 2. Results

### 2.1. Creation of a Data Table

**Figure 1:**
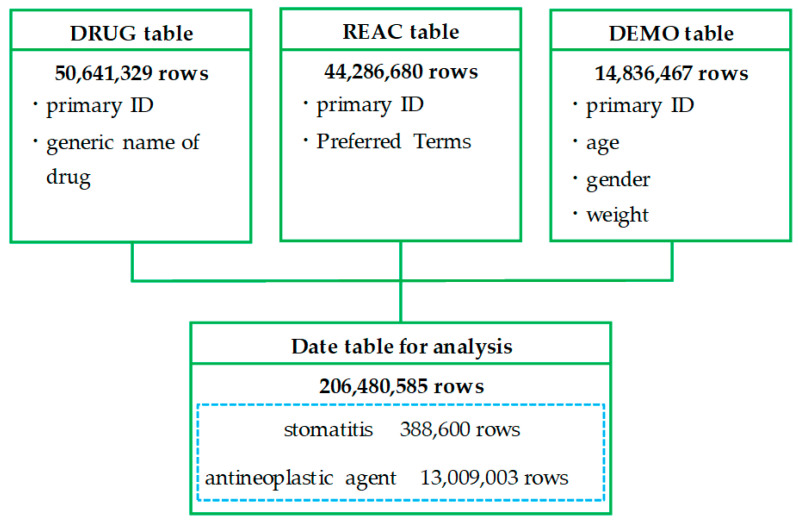
Flowchart of the creation of a data table for analysis. DRUG: drug information, REAC: adverse reaction information, and DEMO: patient demographic information. Duplicate data in the DRUG, REAC, and DEMO tables were deleted and combined using primary IDs.

**Figure 2:**
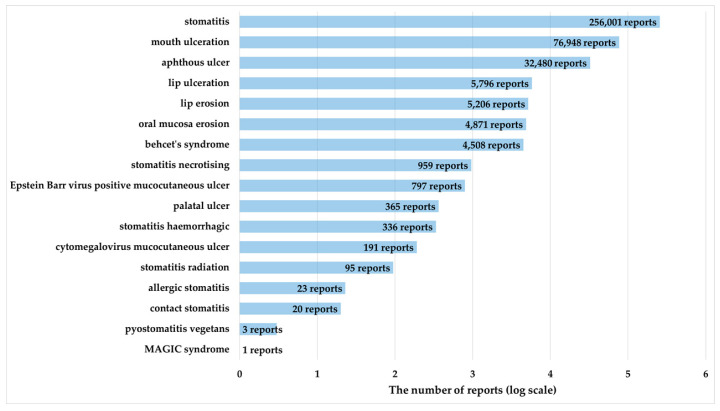
The number of reports with 17 preferred terms related to stomatitis, defined using high-level terms of MedDRA.

Figure 1 shows a flowchart of the process to create the data table for analysis. After the deduplication of each table in the FDA Adverse Event Reporting System (FAERS) database, the drug information (DRUG) table contained 50,641,329 rows, the reaction information (REAC) table contained 44,286,680 rows (22,587 categories), and the demographic and administrative information (DEMO) table contained 14,836,467 rows. The data table for analysis, which was created by combining these tables, contained 206,480,585 rows. Of these, 388,600 rows were for reports on stomatitis, and 13,009,003 rows were for reports on antineoplastic agents. Of the 44,286,680 rows (22,587 categories) of adverse drug events (ADEs) reported to the FAERS, 64,977 rows (17 categories) were preferred terms (PTs) related to stomatitis. Figure 2 shows the number of reports of PTs related to stomatitis defined using high-level terms of MedDRA. Stomatitis was the most frequently reported PT, with 256,001 reports (65.9%).

### 2.2. Association of OM with Patient Characteristics

Deleting unnecessary data from the data table for analysis resulted in 1,322,669 rows, and this table was used to analyze the association between OM and patient gender and age.

Table 1 shows the characteristics of patients with and without stomatitis using antineoplastic agents. Regarding gender, statistically significantly more females were found in the stomatitis group (*p* < 0.0001), with an odds ratio of 0.735 (0.716–0.754) (Table 1). Regarding age, statistically significantly more nonelderly patients (age < 70 years) were in the stomatitis group (*p* < 0.0001), with an odds ratio of 0.907 (0.881–0.932) (Table 1).

### 2.3. Antineoplastic Agents That Induce OM

**Figure 3:**
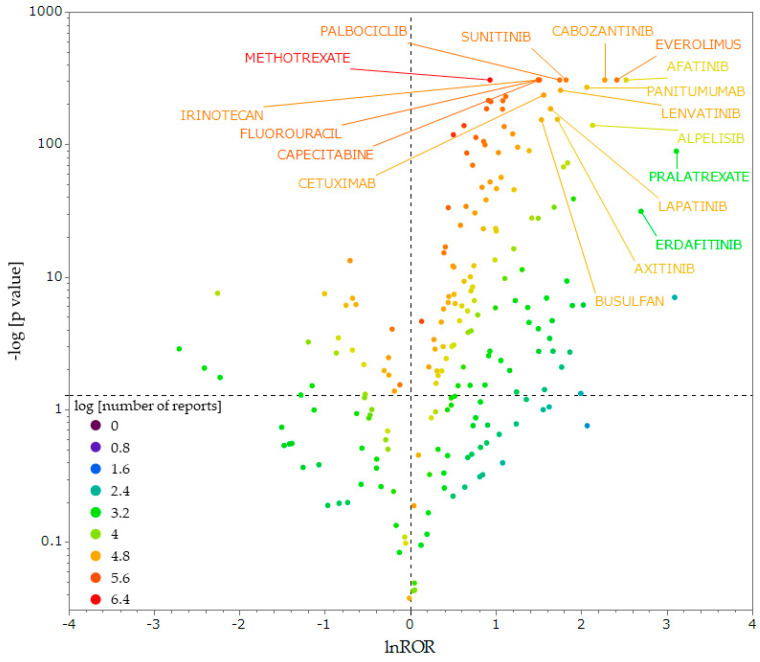
Correlation between antineoplastic agents and stomatitis. The *x*-axis shows lnROR; the *y*-axis shows the ordinary logarithm of the reciprocal *p*-value of Fisher’s exact test (−log [*p*-Value]). The reporting odds ratio (ROR) was calculated from cross-tabulation tables. The dotted line on the *x*-axis indicates lnROR = 0; the dotted line on the *y*-axis indicates *p* = 0.5. The colors in the plots indicate the ordinary logarithm of the total number of adverse events reported for each drug.

Figure 3 is a volcano plot, showing the correlation between antineoplastic agents and stomatitis. Each dot represents an antineoplastic agent, and the larger the natural logarithm of the reporting odds ratio (lnROR) and −log[*p*-value] values, the more likely the drug is to induce stomatitis in a statistically significant manner. We extracted antineoplastic agents with ≥100 reported cases in the data table for analysis and used only reliable signals.

We identified 10 antineoplastic agents, with particular attention to the upper right of the figure (Figure 3). The 10 drugs that showed the most significantly high ROR, termed ROR > 5 and −log10 [*p*-value] > 140, were afatinib, everolimus, cabozantinib, alpelisib, panitumumab, sunitinib, lenvatinib, palbociclib, axitinib, and lapatinib (Table 2). Table 2 shows the ROR, *p*-value, number of reports, Anatomical Therapeutic Chemical Classification System (ATC) code, and ATC name of these antineoplastic agents. All drugs, excluding panitumumab, were classified as “L01E: PROTEIN KINASE INHIBITORS” in the ATC. Panitumumab is an epidermal growth factor receptor inhibitor classified as “L01F: MONOCLONAL ANTIBODIES AND ANTIBODY DRUG CONJUGATES” and are molecular targeted agents. These 10 drugs did not include cell-killing antineoplastic agents such as 5-FU or methotrexate.

### 2.4. MIEs Associated with the Development of Antineoplastic Agent-Induced OM

**Figure 4:**
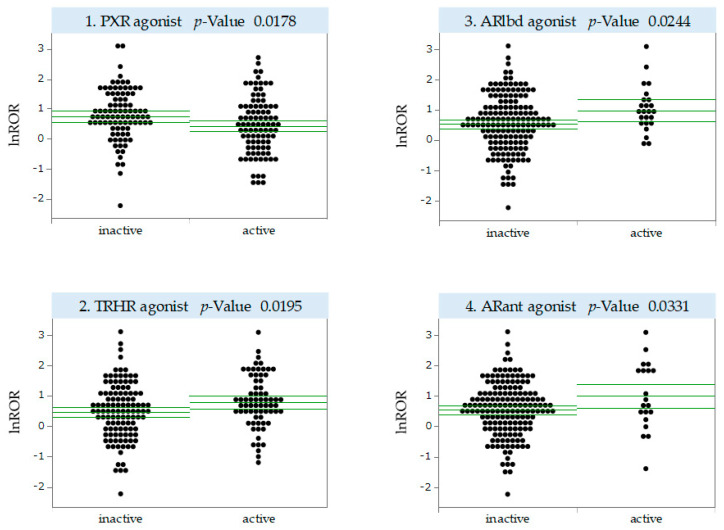
MIEs associated with OM. The vertical axis shows lnROR and the horizontal axis shows MIE activity or not: MIE activity values < 0.5 indicate no activity with a predictive label of “inactive”, whereas MIE activity values > 0.5 indicate activity with a predictive label of “active”. The green line shows the mean value of lnROR and its 95% confidence interval. Each plot represents the antineoplastic agent under analysis. PXR, human pregnane X receptor; TRHR, thyrotropin-releasing hormone receptor; ARlbd, androgen receptor ligand-binding domain; ARant, androgen receptor with antagonist.

Only drugs with ≥100 reports were included in the analysis to ensure the reliability of ROR. There were 183 small-molecule antineoplastic agents with ≥100 reports in the FAERS. We estimated the MIE activities of these 183 drugs using Toxicity Predictor. We performed a univariate analysis of the correlation between predicted MIE activities and lnROR for stomatitis for the 183 antineoplastic agents analyzed. We analyzed 53 MIEs for which activity could be predicted in Toxicity Predictor (Supplementary Table S1) and found statistically significant results for 4 MIEs, suggesting an association with OM development (Figure 4). In Figure 4, each dot represents an antineoplastic agent. For the human pregnane X receptor (PXR), the mean value of lnROR was significantly smaller in the presence of agonist activity, suggesting a significant suppression of OM development. By contrast, for the thyrotropin-releasing hormone receptor (TRHR), androgen receptor ligand-binding domain (ARlbd), and androgen receptor with antagonist (ARant), the mean value of lnROR was significantly greater in the presence of agonist activity, indicating the potential for significantly inducing OM development (Figure 4).

## 3. Discussion

### 3.1. OM and Patient Characteristics

Many risk factors are associated with OM development, including age and female gender. However, the findings are not consistent, and there is little conclusive evidence [21]. In this study, patients who used antineoplastic agents and had stomatitis were more likely to be female and nonelderly (<70 years of age) compared with patients without stomatitis. In most studies, as in this study, the female gender is associated with an increased risk of OM [22,23,24,25,26,27]. Goldberg et al. showed that female patients are more likely to report OM as a treatment complication compared to male patients [23]. Similarly, Chansky et al. reported that female patients are at increased risk of developing severe mucositis when treated with 5-fluorouracil (5-FU) [24]. However, how gender causes different degrees of OM is unclear [26], and contrary reports exist [28,29,30]. In studies investigating gender and 5-FU toxicity, being female is associated with a higher frequency of several toxic events in addition to OM [27,31]. One possible reason for this is the lower ability of females to clear 5-FU [24,28]. Because this study was a comprehensive analysis of many antineoplastic agents, including 5-FU, it is possible that other factors besides clearance, such as hormonal effects, may be involved [32]. Further detailed studies on this mechanism are needed, and gender-based differences in risk are likely to be an important factor to be considered in individual treatment plans. Additionally, our findings indicate that androgen receptor agonist activity may be involved in inducing OM. The increased sensitivity of the androgen receptor has been linked to the development of insulin resistance in postmenopausal women [33]. Because most women analyzed in this study were considered postmenopausal (mean age: 58.7 years), increased sensitivity may be one factor contributing to the development of OM.

Age is a common risk factor for OM; however, OM has been reported in both younger and older individuals [4,7,23,31,34,35,36,37,38], and evidence on the impact of age on OM risk is inconsistent [22,39]. Some reports indicate that the patient’s age does not correlate with OM incidence [25,28]. In this study, nonelderly patients < 70 years old were significantly associated with OM. Sonis reported that children who typically have a higher rate of basal cell proliferation are three times more likely to develop mucositis than the elderly, who have a slower rate of basal cell proliferation [36]. However, age-related physiologic decline in renal function may contribute to OM development in the elderly [40]. One hypothesis is that the risk of developing OM is higher in children and the elderly and lower in adults, which is a stage in between these life stages. However, in this study, the risk of OM by detailed age stratification could not be identified because the patient group was divided into two groups: those <70 years of age and those ≥70 years of age. Therefore, further studies are warranted on the risk of developing OM by finer age stratification and the cause of OM. It is possible that the nonelderly received more aggressive and intense anticancer therapy than the elderly. This may have influenced OM development and may be a bias in this study.

### 3.2. Antineoplastic Agents That Induce OM

We identified antineoplastic agents prone to induce OM among the drugs reported in the FAERS. The results suggest that molecularly targeted agents, including protein kinase inhibitors such as afatinib and everolimus, and antibody preparations such as panitumumab, are more likely to induce OM than cell-killing antineoplastic agents such as 5-FU and methotrexate. However, as shown in the volcano plot, some molecularly targeted agents exhibited a tendency to suppress OM, suggesting that not all molecularly targeted agents are likely to induce OM. Consistently, a study using an adverse event reporting database detected molecular targeted agents, including everolimus, lapatinib, afatinib, and panitumumab, as antineoplastic agents involved in the development of stomatitis [41]. Several other studies have shown that molecularly targeted agents cause OM [42,43,44]. These agents may induce OM differently than cell-killing chemotherapy, and the mechanisms by which molecularly targeted agents cause OM remain unclear [45]. For molecularly targeted agents such as anti-epidermal growth factor receptor drugs and angiogenesis inhibitors, the association with ulcers has been discussed in several studies. The epidermal growth factor acts as a mitogenic factor and induces the synthesis of mucus and prostaglandins, thereby playing a role in the preservation of mucosal integrity and its rehabilitation. According to one hypothesis, the inhibition of squamous epithelial maturation by anti-epidermal growth factor receptor agents contributes to ulcer development [46]. Angiogenesis inhibitors have been shown to delay wound healing by inhibiting transforming growth factor-β1 activation [42,46], which may also be associated with OM.

Although molecularly targeted agents are designed to target specific molecular pathways in cancer cells, this study suggests that they may also affect normal oral mucosal cells and cause OM. Therefore, adequate prevention and management of OM may be necessary even when molecularly targeted agents are used.

### 3.3. MIEs Associated with Antineoplastic Agent-Induced OM Development

We investigated MIEs associated with the development of OM induced by antineoplastic agents. Of the 53 NR and SR pathways evaluated, we identified three: PXR, TRHR, and androgen receptor (AR). We hypothesize that antineoplastic agents with PXR agonist activity significantly suppressed OM development, whereas antineoplastic agents with TRHR and AR agonist activity significantly induced OM development.

PXR is a key element of the body’s defense to toxic compounds, including xenobiotics, that regulates foreign body metabolism by modulating CYP3A and other genes as well as detoxification and elimination [47]. The functions of PXR are not limited to foreign body sensing but also include roles in the inflammatory response and cell proliferation [48]. Activated PXR regulates many genes implicated in biological changes, transportation, inflammation, and oxidative stress, either directly by binding to genomic regions or indirectly through crosstalk with other transcription factors [49]. Because the generation of reactive oxygen species and inflammatory factors is associated with OM development, it is possible that the suppression of OM development observed in this study was a result of the suppression of these functions by a PXR agonist. According to Zhou et al., PXR activation inhibits NF-κB signaling in vivo, which is involved in the production of proinflammatory cytokines, and a marked increase in chronic inflammatory infiltrates has been observed in the small intestine of PXR knockout mice [50]. This interaction may contribute to the mechanism by which PXR suppresses inflammatory bowel disease (IBD) [51]. Several studies have suggested that PXR activity ameliorates IBD by suppressing NF-κB activity in IBD [52,53,54,55]. Garg et al. found that the activation of PXR weakened NF-κB signaling and inhibited the cytokine-induced expression of myosin light-chain kinase and the activation of c-Jun N-terminal kinase 1/2, which may have alleviated barrier dysfunction in the intestinal epithelium [56]. Because the association between PXR and OM has been postulated in this study, and NF-κB is one of the major inflammatory pathways in OM, one hypothesis is that PXR is involved in OM pathogenesis through NF-κB as in IBD. The mechanism by which PXR activation alleviates IBD symptoms could be considered in treating OM, suggesting that PXR is a potential target in OM treatment. However, PXR is a major mechanism for the development of drug resistance in cancer, which may reduce therapeutic efficacy [57], and more research is needed because our knowledge of the association between PXR and OM is limited.

TRHR is a G-protein-coupled receptor that, upon binding to the tripeptide TRH, acts through phospholipase C to increase intracellular inositol triphosphate [58]. TRH promoted homeostasis as a major neurobiological function [59]. TRHR is expressed in the central nervous system and throughout the peripheral nervous system, as well as in other organs and tissues. In vivo and in vitro evidence supports a homeostatic role for TRH in its interaction with the immune system [60]. The TRH immune system homeostasis hypothesis has been proposed as an extension of the homeostasis hypothesis [60]. The interaction of TRH with the immune system may support the finding of an association between TRHR and OM in this study. However, this interaction has both stimulatory and inhibitory activities in a state-dependent manner, and TRH may exhibit anti-inflammatory effects during inflammation, such as OM [60]. A study by Brod et al. showed that orally ingested TRH has an anti-inflammatory effect [61], which contradicts the results of the present study. Other possible explanations for the results of this study could be increased mucosal blood flow—TRH was shown to increase mucosal blood flow in the stomach through the central nervous system [62] and may have similar effects on the oral mucosa. Increased blood flow may lead to the increased exposure of antineoplastic agents to the oral mucosa and progressive inflammation, resulting in the development or worsening of OM. Other possible indirect effects on hormone balance through TRHR cannot be ruled out. Further investigation is needed to understand the association between OM development and TRHR.

For AR, this study found an association between the agonist activity of ARlbd and ARant and OM, where ARlbd represents the ligand-binding domain of AR, and ARant represents AR in the presence of an antagonist. AR exerts various modulatory effects on the immune system [63]. According to Lai et al., monocyte/macrophage AR inhibited wound healing by promoting local TNF-α production through several mechanisms, amplifying the inflammatory response [64]. Furthermore, AR is expressed in the oral mucosa [65], suggesting that AR is indirectly associated with antineoplastic agent-induced OM through proinflammatory cytokines. This mechanism is only one possible reason why an association between AR agonists and OM was observed in this study, and further studies are needed to clarify the correlation between AR and OM.

Further studies on the function of NR and SR pathways (PXR, TRHR, and AR) in OM could lead to the prevention and management of OM during the clinical use of antineoplastic agents.

### 3.4. Limitations

This study has several limitations. First, spontaneous reporting databases accumulate only cases in which some ADEs have occurred and do not provide information on all patients to whom a drug was administered [66]. Therefore, risks are estimated using RORs, which may introduce bias.

Second, there is a potential for reporting bias, including underreporting and selective reporting [67]. Not all ADEs are reported in the database; only a subset of events that actually occur are recorded. In addition, depending on the severity of ADE, known information, and other factors, certain ADEs may be over-reported, or there may be bias in the selection of ADEs to be reported [66]. These are among the most significant problems of spontaneous reporting databases [67].

Third, the FAERS database may contain missing values or incorrectly entered letters or numbers. In this study, reports containing missing values for gender and age were removed, and analyses were conducted for gender and age.

Fourth, it is difficult to identify the exact cause of an ADE when multiple medications are used [66,68]. In this study, the analysis included information on all drugs, including “primary suspect drug”, “secondary suspect drug”, “concomitant”, and “interacting”. Thus, false-positive signals may have been detected.

Finally, the MIE activity values used in this study are not actual measurements but only predictions calculated by Toxicity Predictor. Therefore, the MIEs suggested to be associated with OM may only be a part of various OM-associated biochemical pathways [69].

## 4. Materials and Methods

### 4.1. FAERS Database

The FAERS database contains adverse event reports, medication error reports, and product quality complaints resulting in adverse events that were submitted to the FDA [70]. Adverse events associated with FDA-approved drugs and biologicals are collected from the United States and other countries, and reports are submitted by healthcare professionals, consumers, and manufacturers. In this study, we downloaded the FAERS database from the official FDA website [70] and used data from the first quarter of 2004 to the first quarter of 2022.

### 4.2. Drugs Analyzed and Definitions of ADEs

The drugs analyzed were the 261 antineoplastic agents listed in the FAERS. Antineoplastic agents were defined as drugs classified as “L01:ANTINEOPLASTIC AGENTS” in “L:ANTINEOPLASTIC AND IMMUNOMODULATING AGENTS” based on the Anatomical Therapeutic Chemical Classification [71]. For ADEs, stomatitis was defined as a PT included in the high-level terms of MedDRA/J version 25.0, “Stomatitis and ulceration” [72]. In this study, the following 17 terms reported in FAERS were included in the analysis: aphthous ulcer (PT code: 10002959), Behcet’s syndrome (PT code: 10004213), lip ulceration (PT code: 10024572), mouth ulceration (PT code: 10028034), stomatitis (PT code: 10042128), stomatitis hemorrhagic (PT code: 10042132), stomatitis necrotizing (PT code: 10042135), stomatitis radiation (PT code: 10042137), lip erosion (PT code: 10051992), oral mucosa erosion (PT code: 10064594), cytomegalovirus mucocutaneous ulcer (PT code: 10065036), contact stomatitis (PT code: 10067510), pyostomatitis vegetans (PT code: 10074068), palatal ulcer (PT code: 10077519), MAGIC syndrome (PT code: 10078132), Epstein–Barr virus-positive mucocutaneous ulcer (PT code: 10079386), and allergic stomatitis (PT code: 10079554)

### 4.3. Creation of a Data Table for Analysis

The FAERS consists of the following seven data tables: (1) demographic (DEMO) table, (2) drug (DRUG) table, (3) reaction (REAC) table, (4) indication (INDI) table, (5) outcome (OUTC) table, (6) report sources (RPSR) table, and (7) therapy (THER) table.

Necessary data were extracted from the DRUG, REAC, and DEMO tables, and duplicates were eliminated. Specifically, “primary ID”, and “generic name of drug” were extracted from the DRUG table; “primary ID” and “Preferred Terms” were extracted from the REAC table; and “primary ID”, “age”, “gender”, and “weight” were extracted from the DEMO table. The three tables were joined by “primary ID” to form a data table for analysis.

### 4.4. Characteristics of Patients with Antineoplastic Agent-Induced OM

Female gender and age have been reported as factors associated with OM development [23]. However, evidence regarding the effect of age specifically on mucositis risk is inconsistent [22]. Therefore, we analyzed whether OM is associated with gender and age. From the data table for analysis, we extracted data in which the drug used was an antineoplastic agent. From there, data regarding the presence of stomatitis, gender, age, and primary ID were extracted, and duplicates were eliminated. Data with insufficient or possibly erroneous information on gender and age were removed, and data cleaning was performed to ensure that age was included in the range of 0–120 years. Gender was divided into “female” and “male” groups, and age was defined as “elderly” for patients aged ≥ 70 years and “nonelderly” for patients aged < 70 years. The patients were divided into two groups according to the presence or absence of stomatitis. Bivariate analyses were performed for the correlation between gender and stomatitis and between age and stomatitis. Odds ratios and *p*-values were calculated. Fisher’s exact test was used to calculate *p*-values.

### 4.5. Calculation of RORs

We detected signals of drugs associated with stomatitis using the data table for analysis. ROR was used to assess risk. From the information in the data table for analysis, a 2 × 2 contingency table consisting of the use of drugs and the presence or absence of stomatitis was created (Table 3). Considering the 2 × 2 contingency table, calculations cannot be performed in cells containing 0, and the estimation becomes unstable when the cell values are small. Therefore, 0.5 was added to all cells as a correction (Haldane–Anscombe correction) [73]. We extracted antineoplastic agents with at least 100 reported cases from the data table for analysis, and only reliable signals were used.

### 4.6. Generation of a Scatter Plot

To visually interpret the correlation between the 230 antineoplastic agents reported as (a + b) ≥ 100 and stomatitis, scatter plots of RORs were generated, and *p*-values were calculated. *p*-values were obtained from Fisher’s exact test. In the scatter plots, ROR was used as lnROR and *p*-value as the reciprocal ordinary logarithm (−log10[*p*-value]). The scatter plots corresponded to the volcano plots, which are used to show trends in gene expression for both statistical and biological significance [74].

### 4.7. MIE Activity Prediction Using QSAR Toxicity Predictor

We predicted the agonist–antagonist activity against 53 NR and SR pathways by using Toxicity Predictor [20], a QSAR system developed based on a prediction model built using information from the Tox21 10K library.

Simplified Molecular Input Line Entry System (SMILES) strings for small-molecule antineoplastic agents were collected from PubChem using Python and mapped to 230 antineoplastic agents with (a + b) ≥ 100. Small-molecule antineoplastic agents with corresponding SMILES strings were extracted, and 183 compounds were included in the analysis. Compounds were submitted to Toxicity Predictor to calculate agonist and antagonist activity values (MIE activity) for the NR and SR pathways. Of the 59 MIEs, 6 were excluded because of their small area under the curve values on the receiver operating characteristic (ROC) curve and low prediction accuracy, and 53 were analyzed. The cutoff value of the predicted MIE activity value was determined based on the Youden index [75] using the ROC curve in the prediction model. MIE activity values were normalized so that the cutoff value was 0.5. Predictive labels for compounds with normalized predictive values > 0.5 were assigned “active”, and predictive labels for compounds with predictive values < 0.5 were assigned “inactive”. In MIEs where measured values existed, measured values were used instead of predicted results (Supplementary Table S2).

### 4.8. Univariate Analysis

We estimated MIEs associated with OM by conducting univariate analysis of lnRORs of antineoplastic agents on stomatitis as response variables and predictive labels of MIE activities as explanatory variables. In univariate analysis, we used *p*-value for *t*-test to extract MIEs for which the difference in the mean value of lnROR with and without MIE activity was statistically significant.

### 4.9. Statistical Analysis

All analyses were performed using JMP Pro 16 software (SAS Institute Inc., Cary, NC, USA). The level of statistical significance was set at 0.05.

## 5. Conclusions

We analyzed a large dataset of spontaneously reported adverse events and identified MIEs associated with antineoplastic agent-induced OM. Our analysis showed that OM was significantly more likely to develop in females and nonelderly patients and more prone to be induced by molecularly targeted agents, including protein kinase inhibitors. Furthermore, the agonist activity of PXR, TRHR, and AR may be associated with antineoplastic agent-induced OM. These findings may help avoid OM-induced risks during drug discovery and prevent OM during the clinical use of antineoplastic agents. Drug selection, to the extent possible, may reduce the risk of developing OM. Additionally, developing concomitant drugs that inhibit the factors identified in this study may contribute to reducing OM induction.

## Figures and Tables

**Table 1:** Characteristics of patients with and without stomatitis using antineoplastic agents.

	Patient Background	Stomatitis (25,115)	Without Stomatitis (1,297,554)	Odds Ratio	95% Confidence Interval	*p*-Value
Sex	Female	16,078	735,315	0.735	0.716−0.754	<0.0001
	Male	9037	562,239			
Age	<70 years old	18,300	919,739	0.907	0.881−0.932	<0.0001
	≥70 years old	6815	377,815			

**Table 2:** Antineoplastic agents closely associated with stomatitis.

Antineoplastic Agents	ROR	95% Confidence Interval	*p*-Value	Number of Reports	ATC Code	ATC Name
Afatinib	12.48	11.44–13.6	<0.0001	23,023	L01EB	EGFR tyrosine kinase inhibitors
Everolimus	11.20	10.85–11.56	<0.0001	191,356	L01EG	mTOR kinase inhibitors
Cabozantinib	9.72	9.26–10.22	<0.0001	89,600	L01EX	Other protein kinase inhibitors
Alpelisib	8.43	7.45–9.54	<0.0001	16,464	L01EM	Pi3K inhibitors
Panitumumab	7.88	7.23–8.59	<0.0001	35,861	L01FE	EGFR inhibitors
Sunitinib	6.18	5.9–6.47	<0.0001	163,120	L01EX	Other protein kinase inhibitors
Lenvatinib	5.79	5.36–6.26	<0.0001	59,076	L01EX	Other protein kinase inhibitors
Palbociclib	5.73	5.5–5.98	<0.0001	211,026	L01EF	CDK inhibitors
Axitinib	5.58	5.06–6.16	<0.0001	38,095	L01EK	VEGFR tyrosine kinase inhibitors
Lapatinib	5.15	4.72–5.62	<0.0001	53,535	L01EH	HER2 tyrosine kinase inhibitors

EGFR, epidermal growth factor receptor; mTOR, mammalian target of rapamycin; Pi3K, phosphatidylinositol-3-kinase; CDK, cyclin-dependent kinase; VEGFR, vascular endothelial growth factor receptor; HER2, human epidermal growth factor receptor 2.

**Table 3:** Cross-tabulation and calculation formula for the RORs of stomatitis.

	Stomatitis	Without Stomatitis
Reports with the suspected medicine	a	b
All other reports	c	d

ROR (reporting odds ratio) = (a/b)/(c/d) = ad/bc.

## Data Availability

Data are contained within the article and Supplementary Materials.

## Supplementary Materials

The following supporting information can be downloaded at: https://www.mdpi.com/article/10.3390/ph17081086/s1, Table S1: Univariate analysis to identify MIEs associated with oral mucositis; Table S2: The MIE activity values using Toxicity Predictor.
